# Acute Sheehan Syndrome With Distinctive Arginine Vasopressin Secretion and Magnetic Resonance Imaging Findings

**DOI:** 10.1210/jcemcr/luaf134

**Published:** 2025-07-11

**Authors:** Yuki Minamoto, Yumiko Sasai, Yui Yamashita, Keiko Yamagami, Ichiro Fujisawa, Naotetsu Kanamoto

**Affiliations:** Department of Endocrinology, Osaka City General Hospital, Osaka 534-0021, Japan; Department of Endocrinology, Osaka City General Hospital, Osaka 534-0021, Japan; Department of Endocrinology, Osaka City General Hospital, Osaka 534-0021, Japan; Department of Endocrinology, Osaka City General Hospital, Osaka 534-0021, Japan; Department of Radiology, Kishiwada City Hospital, Osaka 596-8501, Japan; Department of Endocrinology, Osaka City General Hospital, Osaka 534-0021, Japan

**Keywords:** acute Sheehan syndrome, AVP, AVP deficiency, SIADH

## Abstract

Acute Sheehan syndrome is a rare condition that occurs hours to days post partum and causes hypopituitarism. It may cause hyponatremia due to adrenal insufficiency, and most patients improve with steroid therapy. However, hyponatremia is caused not only by adrenal insufficiency but also by inappropriate secretion of arginine vasopressin (AVP). We report the case of a 30-year-old Japanese primipara with massive postpartum hemorrhage and acute Sheehan syndrome. Hyponatremia developed following hypernatremia soon after the postpartum period; however, it did not improve despite adequate hydrocortisone supplementation. AVP fluctuated based on water balance and magnetic resonance imaging findings, showing distinctive AVP secretion dynamics. Oral 1-desamino-8-D-arginine vasopressin was temporarily needed during the clinical course, after which it was not needed, suggesting that impaired blood flow to the posterior pituitary lobe and its improvement may have contributed to the distinctive AVP secretion dynamics. Therefore, when hyponatremia is not improved despite adequate hydrocortisone supplementation in patients with acute Sheehan syndrome, the syndrome of inappropriate antidiuretic hormone secretion should be considered because distinctive AVP secretion dynamics can occur after severe ischemia of the pituitary gland.

## Introduction

Sheehan syndrome is often diagnosed years after postpartum infarction [1]. Its prevalence has decreased in developed countries due to modern obstetric care; however, it remains an issue in developing countries [1]. Its etiology is suspected to involve ischemia or arterial spasms of the pituitary gland due to massive bleeding, hypotension, or thrombosis of the pituitary artery [1]. According to the severity of pituitary gland damage, patients with Sheehan syndrome present with clinical symptoms ranging from isolated hypopituitarism to panhypopituitarism; however, central arginine vasopressin (AVP) deficiency (AVP-D) is rare [1]. Acute Sheehan syndrome is a rare condition that occurs hours to days post partum and causes severe hypopituitarism. It may lead to hyponatremia due to adrenal insufficiency [2], while inappropriate secretion of AVP also contributes to hyponatremia [3]. We report a case of acute Sheehan syndrome due to massive postpartum hemorrhage with distinctive AVP secretion dynamics based on water balance disorder and magnetic resonance imaging (MRI) findings.

## Case Presentation

A 30-year-old Japanese primipara underwent an emergency cesarean delivery due to early placental abruption at 33 weeks of gestation. However, the patient went into shock 3 hours postoperatively. A contrast-enhanced computed tomography scan revealed massive intra-abdominal hemorrhage, and the patient was transferred to our hospital. Her vital signs improved with continuous blood transfusion, and she underwent surgical hemostasis. The total blood loss was 7000 mL. The first day, she presented with polyuria (300 mL/h), high plasma osmolality (314 mOsm/kg), relatively low urine osmolality (220 mOsm/kg), high serum sodium concentration 153 mEq/L (153 mmol/L) (institutional reference range, 138-145 mEq/L [SI: 138-145 mmol/L]), and undetectable AVP. Central AVP-D was suspected, and intranasal 1-deamino-8-D-arginine vasopressin (DDAVP) at 5 mcg/day was initiated on day 2. The polyuria improved; however, on day 5, the serum sodium concentration decreased to 117 mEq/L (117 mmol/L), and intranasal DDAVP was stopped. Nevertheless, the urinary output was 100 mL/h or less, plasma osmolality was 232 mOsm/kg, and urinary osmolality was 706 mOsm/kg. She was referred to our department on day 6 due to electrolyte abnormalities.

## Diagnostic Assessment

**Figure 1.**
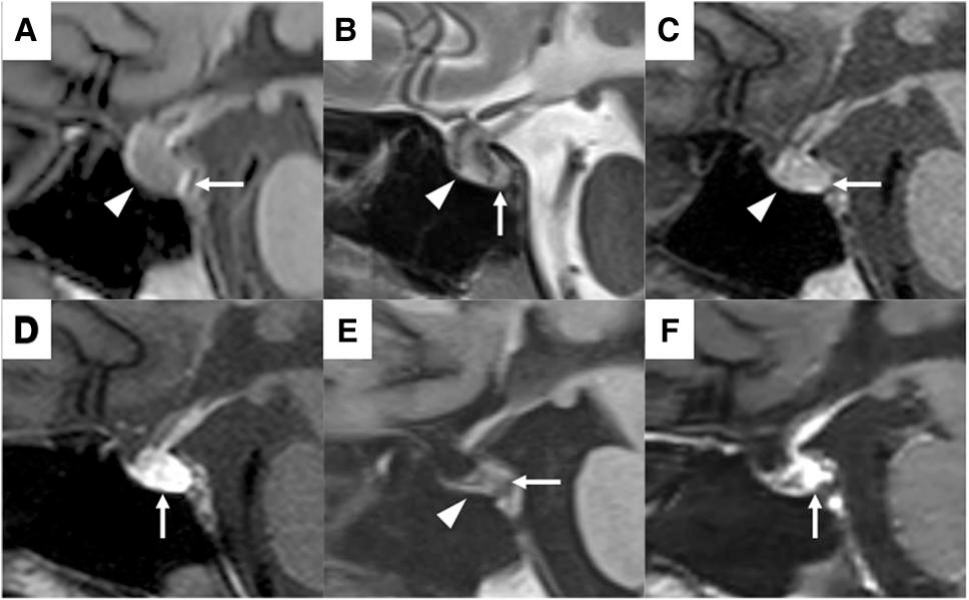
Sagittal MR image of the pituitary. A, T1WI on day 6. The anterior lobe is swollen with a diffuse low signal (arrowhead), and a normal high signal of the posterior lobe is clearly demonstrated (arrow). B, T2WI on day 6. The anterior lobe is demonstrated as an inhomogeneous high signal (arrowhead). The posterior lobe is also demonstrated as a high signal (arrow). C, T1WI on day 12. The swelling of the anterior lobe is improved (arrowhead), and the high signal of the posterior lobe is absent (arrow). D, Contrast enhancement on day 12. The posterior part of the anterior lobe and the whole posterior lobe are enhanced (arrow). E, T1WI on day 45. The anterior lobe is atrophic (arrowhead), and the high signal of the posterior lobe remains absent (arrow). F, Contrast enhancement on day 45. The contrast enhancement in the posterior part of the pituitary lobe is shrunk (arrow).

Abbreviations: MR, magnetic resonance; T1WI, T1-weighted imaging; T2WI, T2-weighted imaging.

**Figure 2.**
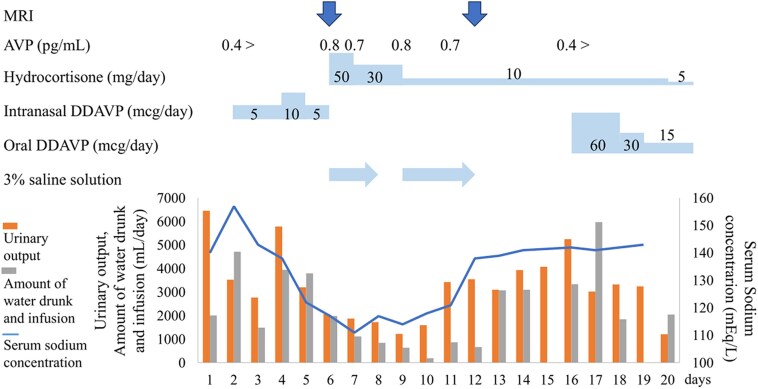
The clinical course of treatment, serum sodium concentration, and water balance. Treatment was based on water balance and serum sodium concentration. The AVP data are listed in the top row.

Abbreviations: AVP, arginine vasopressin; DDAVP, 1-deamino-8-D-arginine vasopressin; MRI, magnetic resonance imaging.

**Figure 3.**
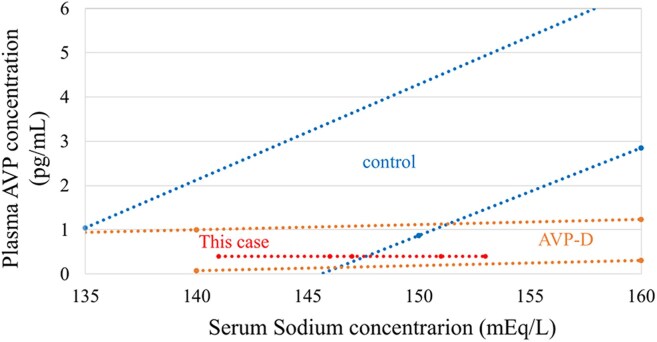
Results of a hypertonic saline tolerance test using the hypertonic saline tolerance assessment tool. The 95% prediction intervals for the plasma AVP concentrations during the hypertonic saline tolerance test are shown by dotted lines for each group [5]. AVP did not increase despite the serum sodium increase, indicating central AVP-D.

Abbreviations: AVP, arginine vasopressin; AVP-D, AVP deficiency.

Notably, the patient had no significant past medical or life history, and her body weight and body mass index were 49.8 kg and 20.0 kg/m^2^, respectively. Her vital signs were stable on day 6. On physical examination, no abnormal heart or lung sounds were observed. Milk secretion was poor, and dehydration or overflow was not suspected. Blood tests showed that AVP was elevated despite hyponatremia, adrenocortical hormone levels were relatively preserved, and other pituitary hormone levels were decreased (Table 1). Hypothyroidism rarely induces hyponatremia; however, it is more likely to occur in cases of severe hypothyroidism [4]. In this case, the hypothyroidism was mild and would not cause hyponatremia. The MRI findings on day 6 showed that the anterior lobe was swollen with a diffuse low signal, and a normal high signal of the posterior lobe was clearly demonstrated on the sagittal T1-weighted image (WI) (Fig. 1A). The whole pituitary gland was demonstrated as an inhomogeneous high signal on T2WI, suggesting infarction of the whole pituitary gland (Fig. 1B). Therefore, based on blood tests, urinary tests, MRI findings, and a history of massive postpartum hemorrhage, we diagnosed acute Sheehan syndrome and suspected hypopituitarism. On day 7, the serum sodium concentration further decreased to 115 mEq/L (115 mmol/L) despite adequate hydrocortisone supplementation, and AVP remained high (Fig. 2), which led us to suspect syndrome of inappropriate antidiuretic hormone secretion (SIADH). On day 11, hypotonic polyuria (3 L/day) was observed. On day 12, serum sodium concentration was elevated to 138 mEq/L (138 mmol/L), and plasma and urine osmolality was 280 mOsm/kg. A rapid increase in serum sodium concentration from 121 mEq/L (121 mmol/L) to 138 mEq/L (138 mmol/L) over one day, accompanied by an increase in hypotonic urine, raised suspicion of recurrent central AVP-D. The MRI findings on day 12 showed that the swelling of the anterior lobe had improved, and the high signal of the posterior lobe was absent on T1WI (Fig. 1C). The posterior part of the pituitary gland was enhanced (Fig. 1D), suggesting the recovery of blood flow. On day 16, AVP was unresponsive in the hypertonic saline tolerance test (Fig. 3) [5], and central AVP-D was diagnosed. In other stimulation tests, panhypopituitarism was diagnosed; however, adrenocorticotropin and cortisol levels remained at baseline (Table 2).

**Table 1.** Laboratory data on day 6

Category	Test	Result	Institutional reference range
Biochemical	Na	117 mEq/L	138-145 mEq/L
		(117 mmol/L)	(138-145 mmol/L)
	Osmolarity	241 mOsm/kg	275-290 mOsm/kg
Urine	Na	244 mEq/L	
		(244 mmol/L)	
	Osmolarity	736 mOsm/kg	50-1300 mOsm/kg
Endocrine	GH	1.16 ng/mL	0.13-9.88 ng/mL
		(1.16 μg/L)	(0.13-9.88 μg/L)
	IGF-1	81 ng/mL	129-304 ng/mL
		(10.6 nmol/L)	(16.9-39.8 nmol/L)
	PRL	20.4 ng/mL	4.9-29.3 ng/mL
		(20.4 μg/L)	(4.9-29.3 μg/L)
	TSH	0.884 μIU/mL	0.5-5.0 μIU/mL
		(0.884 mIU/L)	(0.5-5.0 mIU/L)
	FT3	1.4 pg/mL	2.3-4.0 pg/mL
		(2.2 pmol/L)	(3.5-6.1 pmol/L)
	FT4	0.8 ng/dL	0.9-1.7 ng/dL
		(10.3 pmol/L)	(11.6-21.9 pmol/L)
	ACTH	21.5 pg/mL	7.2-63.3 pg/mL
		(4.7 pmol/L)	(1.6-13.9 pmol/L)
	Cortisol	7.75 μg/dL	7.07-19.6 μg/dL
		(214 nmol/L)	(195-541 nmol/L)
	LH	0.3 > mIU/mL	1.4-15.0 mIU/mL
		(0.3 > IU/L)	(1.4-15.0 IU/L)
	FSH	0.3 > mIU/mL	3.0-10.0 mIU/mL
		(0.3 > IU/L)	(3.0-10.0 IU/L)
	E2	5.0 > pg/mL	28.8-196.8 pg/mL
		(18.4 > pmol/L)	(105.7-722.5 pmol/L)
	Progesterone	0.1 > ng/mL	0.4 > ng/mL
		(0.32 > nmol/L)	(1.27 > nmol/L)
	AVP	0.8 pg/mL	2.8 > pg/mL
		(0.74 pmol/L)	(2.58 > pmol/L)
	NT-proBNP	52 pg/mL	125 > pg/mL
		(6.1 pmol/L)	(14.8 pmol/L)

Abbreviations: ACTH, adrenocorticotropin; AVP, arginine vasopressin; E2, estradiol; Endocrine, endocrine-related tests; FSH, follicle-stimulating hormone; FT3, free triiodothyronine; FT4, free thyroxine; GH, growth hormone; IGF-1, insulin-like growth factor-1; LH, luteinizing hormone; Na, sodium; NT-proBNP, N-terminal pro-B-type natriuretic peptide; PRL, prolactin; TSH, thyrotropin.

**Table 2.** Results of anterior pituitary function tests

Circadian rhythm of hypothalamic-pituitary-adrenal axis*^a^*								
Test	Institutional reference range	7:00	16:00	23:00				
ACTH	7.2-63.3 pg/mL (1.6-13.9 pmol/L)	20.2 pg/mL (4.4 pmol/L)	10.6 pg/mL (2.3 pmol/L)	9.5 pg/mL (2.1 pmol/L)				
Cortisol	7.07-19.6 μg/dL (195-541 nmol/L)	7.25 μg/dL (200 nmol/L)	4.97 μg/dL (137 nmol/L)	4.81 μg/dL (133 nmol/L)				
Urinary free cortisol	4.3-176.0 μg/d (11.9-485.6 μmol/d)	Day 1: 81.7 μg/day (225.4 μmol/d), day 2: 248.0 μg/d (684.2 μmol/d)						
**Insulin tolerance test**								
Test	Institutional reference range	0 min	15 min	30 min	60 min	90 min	120 min	
Glu	73-109 mg/dL (4.05-6.05 mmol/L)	84 mg/dL (4.66 mmol/L)	53 mg/dL (2.94 mmol/L)	26 mg/dL (1.44 mmol/L)	63 mg/dL (3.50 mmol/L)	65 mg/dL (3.61 mmol/L)	72 mg/dL (4.00 mmol/L)	
ACTH	7.2-63.3 pg/mL (1.6-13.9 pmol/L)	20.6 pg/mL (4.5 pmol/L)	18.5 pg/mL (4.1 pmol/L)	34.0 pg/mL (7.5 pmol/L)	48.6 pg/mL (10.7 pmol/L)	30.5 pg/mL (6.7 pmol/L)	33.1 pg/mL (7.3 pmol/L)	
Cortisol	7.07-19.6 μg/dL (195-541 nmol/L)	6.35 μg/dL (175 nmol/L)	6.09 μg/dL (168 nmol/L)	6.08 μg/dL (168 nmol/L)	16.1 μg/dL (444 nmol/L)	11.9 μg/dL (328 nmol/L)	10.9 μg/dL 301 nmol/L)	
GH	0.13-9.88 ng/mL (0.13-9.88 μg/L)	1.35 ng/mL (1.35 μg/L)	0.63 ng/mL (0.63 μg/L)	0.32 ng/mL (0.32 μg/L)	0.80 ng/mL (0.80 μg/L)	1.11 ng/mL (1.11 μg/L)	0.57 ng/mL (0.57 μg/L)	
**GHRP-2 stimulation test**								
Test	Institutional reference range	0 min	15 min	30 min	45 min	60 min		
GH	0.13-9.88 ng/mL (0.13-9.88 μg/L)	0.22 ng/mL (0.22 μg/L)	3.97 ng/mL (3.97 μg/L)	3.76 ng/mL (3.76 μg/L)	2.31 ng/mL (2.31 μg/L)	1.18 ng/mL (1.18 μg/L)		
**TRH stimulation test**								
Test	Institutional reference range	0 min	15 min	30 min	60 min	90 min	120 min	180 min
PRL	4.9-29.3 ng/mL (4.9-29.3 μg/L)	28.0 ng/mL (28.0 μg/L)	38.7 ng/mL (38.7 μg/L)	41.8 ng/mL (41.8 μg/L)	30.7 ng/mL (30.7 μg/L)	29.1 ng/mL (29.1 μg/L)	28.7 ng/mL (28.7 μg/L)	N/A
TSH	0.5-5.0 μIU/mL (0.5-5.0 mIU/L)	2.11 μIU/mL (2.11 mIU/L)	5.35 μIU/mL (5.35 mIU/L)	5.73 μIU/mL (5.73 mIU/L)	4.21 μIU/mL (4.21 mIU/L)	3.44 μIU/mL (3.44 mIU/L)	2.86 μIU/mL (2.86 mIU/L)	N/A
T3	80-160 ng/dL (1.23-2.46 nmol/L)	105 ng/dL (1.61 nmol/L)	N/A	N/A	114 ng/dL (1.75 nmol/L)	N/A	123 ng/dL (1.89 nmol/L)	117 ng/dL (1.80 nmol/L)
**LHRH stimulation test**								
Test	Institutional reference range	0 min	15 min	30 min	60 min	90 min	120 min	
LH	1.4-15.0 mIU/mL(1.4-15.0 IU/L)	1.6 mIU/mL (1.6 IU/L)	3.3 mIU/mL (3.3 IU/L)	3.7 mIU/mL (3.7 IU/L)	3.7 mIU/mL (3.7 IU/L)	3.3 mIU/mL (3.3 IU/L)	3.4 mIU/mL (3.4 IU/L)	
FSH	3.0-10.0 mIU/mL (3.0-10.0 IU/L)	5.1 mIU/mL (5.1 IU/L)	6.4 mIU/mL (6.4 IU/L)	7.2 mIU/mL (7.2 IU/L)	7.4 mIU/mL (7.4 IU/L)	7.3 mIU/mL (7.3 IU/L)	7.7 mIU/mL (7.7 IU/L)	

Abbreviations: ACTH, adrenocorticotropin; FSH, follicle-stimulating hormone; GHRP-2, pralmorelin hydrochloride; GH, growth hormone; Glu, glucose; LH, luteinizing hormone; LHRH, luteinizing hormone–releasing hormone; N/A, not applicable; PRL, prolactin; T3, triiodothyronine; TRH, thyrotropin-releasing hormone; TSH, thyrotropin.

^*a*^A total of 10 mg of hydrocortisone was administered in the morning.

## Treatment

Considering the possibility of adrenal insufficiency, hydrocortisone treatment was initiated on day 6. However, hyponatremia progressed, and urinary output and AVP did not change. This led us to suspect that there was SIADH rather than adrenal insufficiency. Hyponatremia was corrected with 3% saline and an oral water intake restriction of 500 mL/day. On day 11, hypotonic polyuria occurred, and the serum sodium concentration improved rapidly, so the 3% saline and oral water intake restrictions were stopped. On day 16, after central AVP-D diagnosis, we initiated and adjusted for oral DDAVP. The patient's general condition improved, and she was discharged with a prescription of hydrocortisone 5 mg and oral DDAVP 15 mcg on day 20 (see Fig. 2).

## Outcome and Follow-up

Notably, hyponatremia due to free water retention from oral DDAVP was suspected on day 27. Therefore, oral DDAVP was discontinued, and polyuria did not recur. However, the MRI findings on day 45 showed that the anterior lobe was atrophic and that the high signal of the posterior lobe remained absent on T1WI (Fig. 1E), inferring the presence of partial central AVP-D. The contrast enhancement in the posterior part of the pituitary gland had shrunk (Fig. 1F). Regarding the anterior pituitary function, early-morning cortisol levels remained at the same level as before (7.0 μg/dL [193 nmol/L]; institutional reference range, 7.07-19.6 μg/dL [SI: 194-541 nmol/L]), and hydrocortisone was continued at 5 mg. Milk secretion was poor, menstruation started on day 162, and growth hormone replacement started on day 451.

## Discussion

We report a case of acute Sheehan syndrome secondary to massive postpartum hemorrhage. The course of this patient suggested two critical clinical issues. Distinctive AVP secretion dynamics corresponding to the water balance were observed during the clinical course. MRI findings were useful to exclude differential diagnoses. A case of pituitary apoplexy with SIADH and subsequent central AVP-D has been reported previously [6]. However, our case is unique in that it demonstrated a characteristic AVP secretion pattern by measuring AVP in addition to sodium and osmolarity.

Distinctive AVP secretion dynamics corresponding to the water balance were observed during the clinical course. Occasionally, an AVP triphasic secretion pattern can occur after transsphenoidal pituitary surgery. In these cases, a second central AVP-D may become persistent [7 ]. This case differs from previous reports in that blood flow was impaired due to massive hemorrhage and hypotension. Most of the anterior pituitary lobe receives blood from the long portal vein; its posterior part receives blood from the short portal vein. The posterior pituitary lobe receives blood from the inferior hypophyseal artery (IHA), a direct branch of the internal carotid artery. The IHA supplies blood to the short portal vein. MRI findings demonstrated that the severe ischemic changes occurred in the entire pituitary gland, which later became atrophic. The enhancement of the posterior part of the pituitary gland on day 12 suggests that the blood flow recovered mainly from the IHA [8].

AVP is synthesized in the cell bodies of magnocellular neurons located in the paraventricular and supraoptic nuclei in the hypothalamus, enveloped by neurosecretory granules, which are transported by axonal flow, and stored in the posterior lobe. A stimulus that promotes AVP secretion acts on the cell body of the magnocellular neuron, where an action potential is generated and propagated along the long axon to the posterior lobe. AVP is released into the bloodstream by exocytosis promoted by this action potential. The posterior lobe is demonstrated as a characteristic high signal on T1WI, which reflects the normal vasopressin storage and disappears in AVP-D. Interestingly, the normal high signal on T1WI was present despite AVP-D in this case. We suggest that the action potential did not reach the posterior pituitary lobe due to ischemic change. Brain ischemia triggers acute mitochondrial damage and a local energy crisis, leading to degeneration [9]. The neuronal structures of the cerebral cortex can be reversibly restored when the duration of ischemia is less than 1 hour. However, irreversible and progressive neuronal damage occurs with a longer duration of ischemia [10 ].

**Figure 4.**
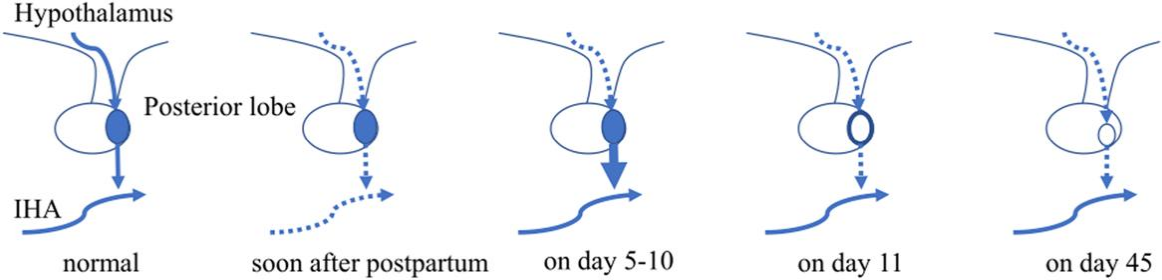
The mechanism of distinctive AVP secretion dynamics in this case. The IHA became ischemic when the patient went into shock soon after post partum, and central AVP-D developed, although AVP had accumulated in the posterior pituitary lobe. On day 5, IHA flow had recovered, and the degeneration of the posterior pituitary lobe due to ischemia caused excess AVP, leading to SIADH. However, the neuronal damage remained. On day 11, the AVP in the posterior pituitary gland had dried up, and central AVP-D developed again.

Abbreviations: AVP, arginine vasopressin; AVP-D, AVP deficiency; IHA, inferior hypophyseal artery; SIADH, syndrome of inappropriate antidiuretic hormone secretion.

In this case, although the duration of ischemia of the pituitary gland and the degree of impairment of the posterior pituitary gland were unknown, distinctive AVP secretion dynamics may have occurred as follows: 1) ischemia of the posterior pituitary lobe and absence of AVP release from the nerve endings, probably due to disturbance in the transmission of action potentials, caused central AVP-D (soon after post partum); 2) degeneration of the posterior pituitary lobe caused excess AVP secretion, resulting in SIADH (days 5-10); 3) SIADH discontinued when AVP storage in the posterior pituitary lobe was depleted, probably due to lack of recovery from the degeneration of the magnocellular neuron (day 11); and 4) central AVP-D occurred again and persisted (day 12 onward) (Fig. 4). The triphasic AVP secretion pattern is quite similar to that observed after transsphenoidal pituitary surgery. However, the mechanism has not been fully understood. The characteristic MRI findings in this case have not been reported in the literature and might provide some important clues to its elucidation.

MRI findings were useful to exclude differential diagnoses. Notably, lymphocytic hypophysitis or apoplexy associated with pituitary tumors is also a cause of postpartum hypopituitarism [1]. On contrast-enhanced MRI, lymphocytic hypophysitis is characterized by homogeneous enhancement of the anterior pituitary gland [1]. Apoplexy associated with pituitary tumors is characterized by enlargement of the sella turcica, erosion of the sellar floor, lateral deviation of the pituitary stalk, contrast enhancement of the periphery, and the persistent presence of a mass near the pituitary gland on repeated imaging [1 ].

In conclusion, we report a case of acute Sheehan syndrome due to massive postpartum hemorrhage. Distinctive AVP secretion dynamics corresponding to water balance were observed during the clinical course, and MRI findings were useful to exclude differential diagnoses. Distinctive AVP secretion dynamics can occur not only after transsphenoidal pituitary surgery but also after severe ischemia of the pituitary gland. When hyponatremia occurs in patients with acute Sheehan syndrome, in addition to adrenal insufficiency, SIADH should be considered, and patients should be carefully monitored.

## Learning Points

- Acute Sheehan syndrome should be considered when hyponatremia occurs in patients with massive hemorrhage and hypotension at birth.
- Hyponatremia can be caused by adrenal insufficiency and SIADH and should be treated cautiously.
- Distinctive AVP secretion dynamics may occur depending on the degree of ischemia of the pituitary gland and degeneration of neurons, and the patient should be carefully monitored because central AVP-D may be relieved later.
- Contrast-enhanced MRI performed early in the course of Sheehan syndrome may help differentiate it from other diseases and determine its severity.

## Data Availability

The original data generated and analyzed during this study are included in this published article or the data repositories listed in “References.”

## Abbreviations

AVP: arginine vasopressin
AVP-D: arginine vasopressin deficiency
DDAVP: 1-deamino-8-D-arginine vasopressin
IHA: inferior hypophyseal artery
MRI: magnetic resonance imaging
SIADH: syndrome of inappropriate antidiuretic hormone secretion
WI: weighted imaging
